# Antibody-mediated targeting of cleavage-specific OPN-T cell interactions

**DOI:** 10.1371/journal.pone.0214938

**Published:** 2019-04-05

**Authors:** Bettina Wanko, Matteo Tardelli, Alexander Jürets, Angelika Neuhofer, Gerhard Prager, John Morser, Lawrence L. Leung, Günther Staffler, Maximilian Zeyda, Thomas M. Stulnig

**Affiliations:** 1 Clinical Division of Endocrinology and Metabolism, Department of Medicine III, Medical University Vienna, Vienna, Austria; 2 Division of Hematology, Stanford University School of Medicine, Stanford, California, United States of America; 3 Veterans Affairs Palo Alto Health Care System, Palo Alto, California, United States of America; 4 Division of Gastroenterology and Hepatology, Department of Medicine III, Medical University of Vienna, Vienna, Austria; 5 Department of Surgery, Medical University Vienna, Vienna, Austria; 6 AFFiRiS AG, Vienna, Austria; Forsyth Institute, UNITED STATES

## Abstract

T cells are crucial players in obesity-mediated adipose tissue inflammation. We hypothesized that osteopontin (OPN), an inflammatory protein with enhanced activity when proteolytically cleaved, affects the number of viable T cells in adipose tissue and assessed inhibition of the interaction between T cells and thrombin and matrix metalloproteinases-cleaved OPN using antibodies and postimmune sera. Gene expression of T cell markers in adipose tissue from wild-type (wt) and *Spp1*^*-/-*^ (OPN deficient) mice was analyzed after 16 weeks of high fat diet (HFD) or low fat diet (LFD) feeding. CD3, CD8 and OPN gene expression in omental adipose tissue from individuals with obesity was measured. OPN-T cell interactions were assessed with a fluorescence-based adhesion assay and blocked with antibodies targeting OPN. Comparison of T cell gene expression in adipose tissue from wt and *Spp1*^*-/-*^ mice showed that OPN affected the number of T cells while in humans, levels of OPN correlated with T cell markers in omental adipose tissue. The interaction between T cells and cleaved OPN was blocked by postimmune sera following OPN peptide vaccinations and with monoclonal antibodies. In conclusion, levels of OPN affected the number of T cells in obesity and antibodies against cleaved OPN antagonize OPN-T cell interactions.

## Introduction

T cells, mostly Th1 cells [1] as well as CD8+ T cells (cytotoxic T cells) [2], play an important role in obesity-mediated adipose tissue inflammation as they infiltrate adipose tissue at an early stage of inflammation[1–3]. Interferon γ (IFNγ), secreted by Th1 and CD8+ T cells, triggers the polarization of macrophages towards a M1 phenotype while the Th2-secreted cytokines IL-4 and IL-13 induce a shift towards a M2 phenotype[4]. Depletion of CD8, either by genetic ablation or antibodies, reduces the number of macrophages in adipose tissue while increasing insulin sensitivity [2]. Passive vaccination with an anti-CD3 antibody or its F(ab’)_2_ fragment improves obesity-induced insulin resistance and reduces the number of M1 type macrophages in adipose tissue [1,5]. Hence, T cells are crucially involved in the initiation of obesity-driven adipose-tissue inflammation and its metabolic sequelae.

OPN (secreted phosphoprotein 1, SPP1), a matricellular protein that acts as a cytokine, is highly upregulated in adipose tissue in obesity [6]. In diet-induced obesity (DIO) models, OPN recruits macrophages into adipose tissue [6]. Active thrombin and matrix metalloproteinases (MMP) cleave OPN [7,8], leading to exposure of otherwise cryptic integrin binding domains enhancing the bioactivity of OPN [9,10]. MMP-cleaved (MMP-cOPN) [11] and thrombin-cleaved OPN (Thr-cOPN) are both involved in the pathogenesis of various diseases including experimental autoimmune encephalomyelitis (EAE) [12], rheumatoid arthritis and glioblastoma [13,14].

To test if OPN affects the number of T cells in adipose tissue, we fed wt and OPN deficient (*Spp1*^*-/-*^) mice high-fat (HFD) or low-fat diet (LFD) and analyzed gene expression of T cell markers in adipose tissue. Our results suggest that OPN might contribute to T cell levels in adipose tissue and our immunotherapy targeting OPN-T cell interactions envisions a novel therapy to treat obesity-associated diseases.

## Methods

### Ethics statement

The described study included human volunteers and was performed according to the principles outlined in the Declaration of Helsinki and Good Clinical Practice Guidelines at the Department of Medicine III, Medical University of Vienna. It was approved by the Ethics committee of the Medical University of Vienna (EK no. 1241/2015 and 074/2008). All patients gave written informed consent.

The study on animals was approved by the Institutional Animal Care and Use Committee (IACUC) of the VA Palo Alto Health Care System (protocol #1566). Animals were housed at the VA Palo Alto Health Care System (VAPAHCS) under a 12h light/dark cycle with free access to food and water. Animal experiments adhered to the 3 Rs of animal welfare (Replacement, Reduction and Refinement).

### Animal experiments

Male B6.Cg-Spp1^tm1Blh^/J (*Spp1*^*-/-*^) mice (OPN deficient mice) and C57BL/6J (wt) were both from Jackson Laboratory (Sacramento, CA) and the mice were fed irradiated HFD (60 kcal% fat, D12492i, Research Diets, New Brunswick, NJ) or irradiated LFD (10 kcal% fat, D12450Bi, Research Diets) from the age of 7 weeks for 16 weeks. The number of animals per group was determined by power calculation (wt mice on LFD: n = 24, wt mice on HFD: n = 26, *Spp1*^*-/-*^ mice on HFD: n = 19). For the power calculation to determine group size, the online tool available at http://www.stat.ubc.ca/~rollin/stats/ssize/n2.html, employing a 2 sided test was used with data from previous experiments.

Water was changed twice a week, HFD twice a week and LFD once a week. Mice were fed ad libitum. Up to 4 mice were housed in one cage supplied with environmental enrichment under specific pathogen free (SPF) condition. *Spp1*^*-/-*^ and wt mice on HFD had the same weight at the age of 7 weeks (wt LFD: 22.9 g ± 0.31 (SEM), wt HFD: 22.94g ± 0.27 and *Spp1*^*-/-*^ mice: 24.4g ± 0.66)and when they were sacrificed (wt LFD: 32.8g ± 0.78, wt HFD: 50.13g ± 0.65, *Spp1*^*-/-*^ mice: 48.3g ± 1.3), with no statistically significant difference between *Spp1*^*-/-*^ mice and wt mice on HFD (p = 0.2825) but different from wt LFD (p<0.0001) (2-way ANOVA and Dunnett Post Hoc test). This is in agreement with published findings [6,15,16]. Mice were sacrificed by CO_2_ inhalation.

### Gene expression

RNA from mouse gonadal fat and human omental fat was extracted with Trizol (Thermo Scientific, Waltham, MA, USA) before cDNA was synthesized using the M-MLV Reverse Transcriptase kit (Promega, Madison, WI). Gene expression was normalized to ubiquitin and analyzed by quantitative real-time polymerase chain reaction (RT-PCR) using GoTaq Probe qPCR mastermix (Promega) and TaqMan primers according to the manufacturer’s protocol. RT-PCR results were quantified using the 2-^ΔΔCT^ method with the LFD treated group set to 100% in case of the RT-PCRs using mouse samples. The Taqman primers used were: CD8a, Mm01182108_m1; CD3a, Mm01179194_m1; CD4, Mm00442754_m1; Ubc, Mm01201237_m1; Ubc, Hs00824723_m1; CD3a, Hs99999153_m1; CD8a, Hs00233520_m1; Spp1, Hs00959010_m1; GATA3, Hs00231122_m1; Tbet, Hs00203436_m1; Foxp3, Hs01085832_m1: Life Technologies, Carlsbad, CA, USA.

### Tissue staining

Formalin-fixed adipose tissue sections were de-paraffinized and blocked for 1 h in 3% goat serum (Dako). Afterwards, slides were incubated at RT for 1 h with monoclonal rat anti mouse CD8 antibody (Abcam, Cambridge, UK) washed with PBS and incubated at RT for 1 h with monoclonal rabbit anti-mouse CD3 antibody (Abcam). Slides were then washed and incubated with the respective secondary antibody: Alexa Fluor 647 goat anti rat IgG for CD8 and Alexa Fluor 488 goat anti rabbit IgG for CD3 (both Thermo Fisher Scientific, Waltham, Massachusetts, USA). For CD4 staining slides were incubated at RT for 1 h with Alexa 488 goat anti mouse CD4 antibody (Biolegend). Nuclei were counterstained with DAPI (Sigma-Aldrich) and mounted (VECTASHIELD Mounting Medium, Vector Laboratories, CA, USA) for microscopic analysis (Olympus BX51) using the 10x objective. Inserts are shown in 40x magnification. Relative quantification of immunohistochemistry staining was performed using Image J software in an automated fashion. using a method adapted from [17,18]. In brief, channels were split for the respective fluorochrome, and total fluorescence was quantified using macros in parallel for each of the pictures taken. The number of CD3/4/8 cells was divided by the number of nuclei per field. 2 to 5 photomicrographs were taken for each slide for quantification. Data are presented as mean values ± standard error (SEM)

### Patients

Omental fat samples were taken from patients with morbid obesity (BMI 39–73 kg/m^2^) undergoing laparoscopic bariatric surgery as well as lean and overweight patients (BMI 19–29.9) undergoing other laparoscopic surgery. Human omental fat samples were isolated as previously described [19].

### Isolation of human T cells from blood

PBMCs were isolated from the blood of healthy volunteers. The ethics committee of the Medical University of Vienna approved this study and all volunteers gave informed consent. After Ficoll density centrifugation T cells were isolated using the Pan T cell isolation kit (Miltenyi Biotech, Bergisch Gladbach, Germany).

### Cell viability assay

In 3 independent experiments, 10^6^ primary human T cells from 3 donors were activated with 2 μg/ml PHA (Sigma, Darmstadt, Germany) in serum free RPMI medium and stimulated with 37.5 nM, 75 nM, 150 nM and 300 nM recombinant full length (FL)-OPN, Thr-cOPN, MMP-cOPN [20]. Additionally, GST-OPN-CTF (C-terminal fragment) was tested in one experiment using T cells from 3 donors. [21]. As controls, cells were stimulated with BSA and without OPN (negative control). Additionally, as a positive control, cells were cultured in medium with 10% FCS. After 72 h incubation at 37°C, the CellTiter-Glo Luminescent Cell Viability Assay (Promega) was used according to the manufacturer’s protocol and the released ATP was taken as a measure of cell viability [22].

### Monoclonal antibodies

Monoclonal antibodies recognizing human OPN were produced as previously described [20]. Antibody 9–3 was raised against peptide C-GDSVVYG while antibody 21–5 was raised against peptide C-TYDGRGDSVVYG-CO-NH_2_.

An IgG1 isotype control was used (M5284, Sigma) for *in vitro* experiments.

### Postimmune sera

Peptides were chosen from the central integrin binding region of OPN. Peptide 1, C-TYDGRGDSVVYG-CO-NH2 represents the C-terminus of MMP-cOPN, peptide 2, C-VVYGLR-COO, represents the C-terminus of Thr-cOPN, peptide 3, C-RGDSVVYG-COO, represents the C-terminus of the MMP-cOPN and a scrambled control peptide from the whole of the region, C-SGRVYGDLVGRD-CO-NH2, were synthesized (EMC microcollections, Tübingen, Germany). Vaccination experiments were performed[20] by coupling the peptides to KLH and injecting the antigens into C57BL/6J mice (30 μg peptide, subcutaneously) at biweekly intervals with alum (Brenntag, Mülheim, Germany) as an adjuvant. 2 weeks after the fourth vaccination, mice were sacrificed and the postimmune serum was analyzed. Sera from 5 mice injected with the same peptide were pooled. Due to limited availability of the different sera, this experiment was only performed once with each point in the *in vitro* cell binding assays being duplicated.

### Cell adhesion assay

Adherence of cells to OPN was measured in a V-well based assay using fluorescently labeled cells. Human T cells (primary human T cells and Jurkat T cells [ATCC, Middlesex, UK]) were labeled with 1 μM CMFDA (Molecular Probes, Eugene, OR, USA) in Jurkat integrin activation medium (DMEM without phenol red [Gibco, Waltham, MA, USA] + 10% FCS ([Merck, Darmstadt, Germany]) + 0.2 mM MnCl_2_) or primary T cell activation medium (DMEM without phenol red [Gibco] + 10% FCS [Merck] + 5 ng/ml phorbol-12-myristate-13-acetate (PMA) + 2 mM MnCl_2_ + 2 mM MgCl_2_ + 500 ng/ml A23817 [Sigma]) for 30 min at 37°C under constant agitation (700 rpm) [20,23]. Labelled cells were washed twice in either Jurkat or primary T cell activation medium by centrifugation at 300 g for 5 min followed by resuspension in the same medium at 10^6^ cells/ml. Various amounts of OPN (FL and cleaved OPN) were coated onto V-shaped microtiter plates (Greiner bio-one, Kremsmünster, Austria) in 50 μl coating buffer (20 mM Tris, 15 mM NaCl, pH 9.4) for 1 h at 37°C. As a no binding control, some wells were not coated with OPN but would be coated with BSA in the subsequent blocking step. The assay was run in duplicate. After blocking non-specific sites with 100 μl/well of 1% BSA in PBS for 1 h at 37°C, plates were washed twice with PBS and incubated with antibodies or postimmune sera (50 μl/well, 1 h 37°C), diluted in PBS. Plates were washed twice with PBS before addition of 50 μl labelled T cells (50.000 cells/well) for 1 h at 37°C. Plates were then centrifuged (30 g, 5 min, 4°C) and measured using an EnSpire Multimode Reader (Perkin Elmer, Waltham, MA, USA) in bottom reading mode (excitation 490 nm, emission 520 nm). The percentage of T cells adhering to OPN was calculated according to Jurets et al. [20].

### Statistics

Groups were compared by ANOVA followed by the Dunnett Post Hoc Test. Correlations were calculated by the Spearman method. P values of <0.05 were accepted as significant.

## Results

### Deficiency of OPN reduced expression of T cell markers in gonadal adipose tissue of obese mice

**Fig 1:**
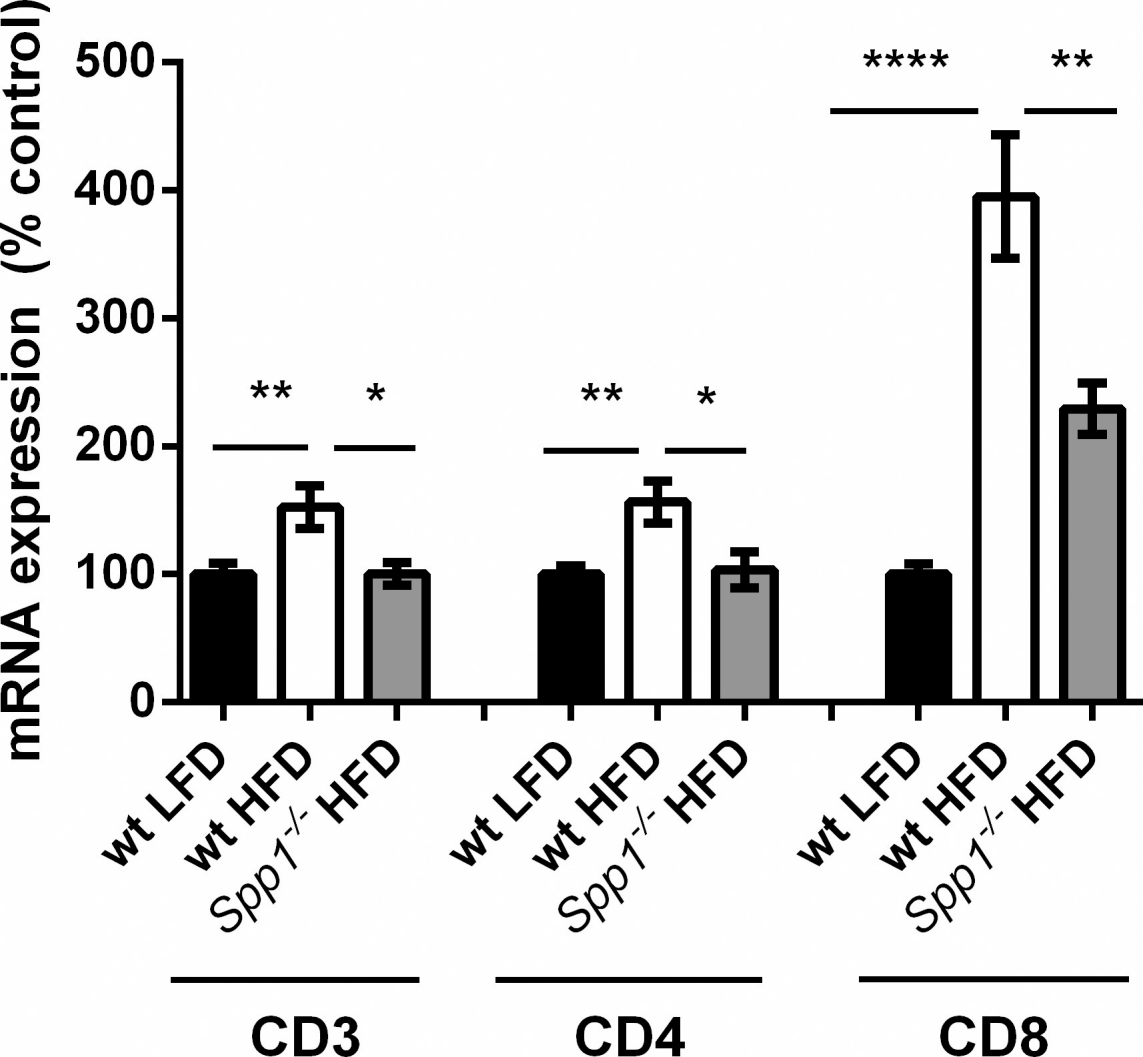
OPN affects T cell numbers in mouse. wt (n = 26) and *Spp1*^*-/-*^ mice (n = 19) were fed HFD or wt were fed LFD (n = 24) for 16 weeks. Gonadal fat was isolated and analyzed by RT-PCR for expression of CD3, CD4 and CD8; mean ± SEM depicted.

To determine the impact of OPN on adipose tissue inflammation in obese mice, wt and *Spp1*^*-/-*^ mice were fed with either HFD or LFD for 16 weeks. After sacrifice, gonadal fat was isolated and T cell marker gene expression was analyzed by RT-PCR. HFD fed wt mice had an increase in CD3, CD4 and CD8 gene expression in adipose tissue compared to LFD fed wt mice (Fig 1). Most pronounced was the ~4 fold upregulation of CD8 gene expression. Compared to wt mice, HFD fed *Spp1*^*-/-*^ mice exhibited a marked reduction in T cell marker gene expression with CD8 gene expression reduced by approximately ~50% suggesting that lack of OPN reduces the number of T cells in adipose tissue in HFD fed mice. The change in expression of these markers could also be due to reductions in other cells that express CD8 apart from T cells.

### The number of T cells is reduced in adipose tissue of *Spp1*^*-/-*^ mice

**Fig 2:**
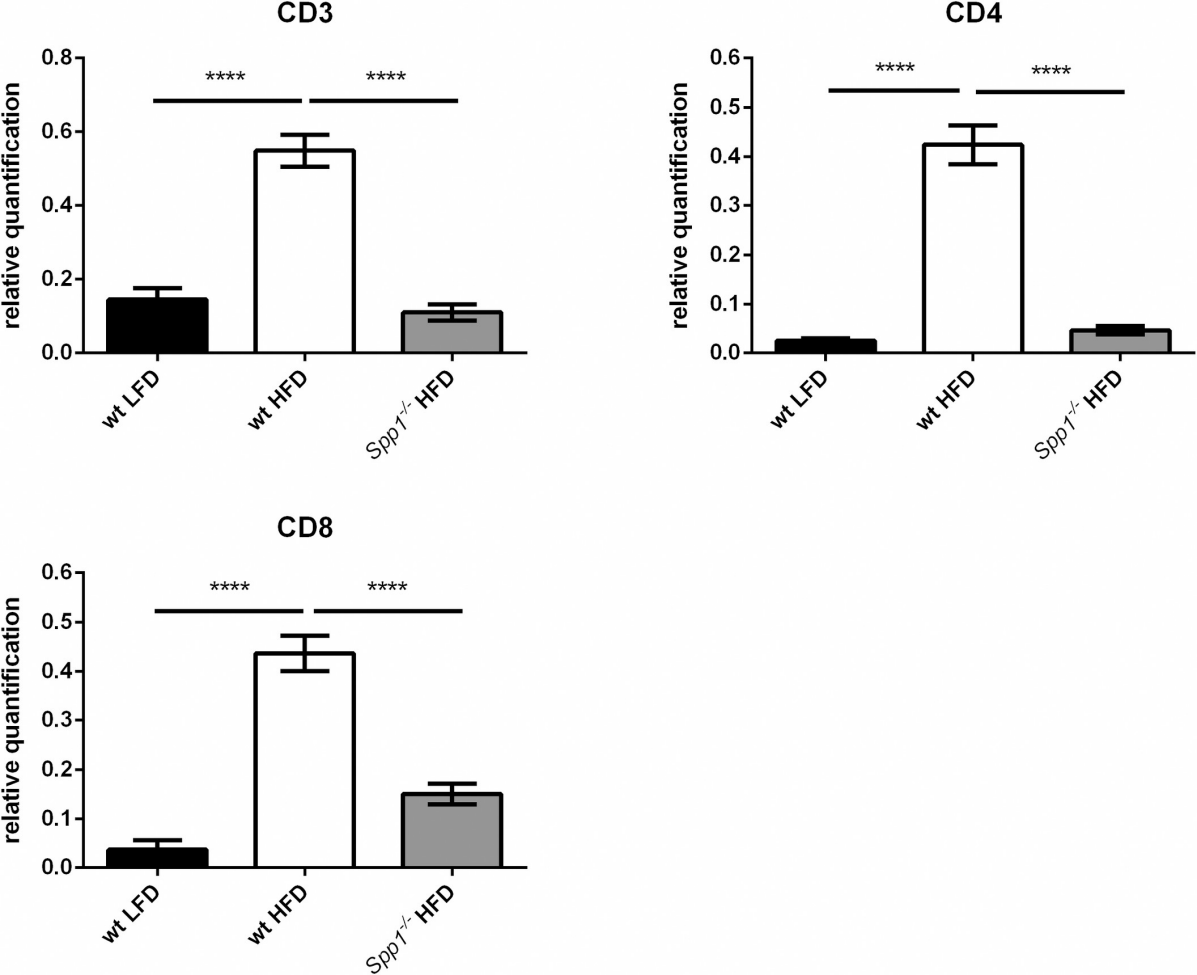
Relative amount of T cells in adipose tissue. The CD3, CD4 and CD8 immunofluorescence intensity in gonadal fat was measured and divided by the number of nuclei (stained with DAPI) resulting in a relative quantification. 2–5 pictures were taken per mouse, 4–5 mice were analyzed. Comparison of groups with wt HFD group; **** p < 0.0001.

The gonadal fat of sacrificed mice was paraffin embedded and stained by immunofluorescence for the T cell markers CD3, CD4 and CD8 (S1 Fig). Relative quantification of the number of CD3+, CD4+ and CD8+ cells (Fig 2) showed that the number of CD3+, CD4+ and CD8+ cells was significantly higher in wt mice fed HFD compared to wt mice fed LFD. There was a highly significant reduction in T cells in adipose tissue of *Spp1*^*-/-*^ mice which strongly suggests that OPN is implicated in regulating the number of T cells in adipose tissue.

### OPN and T cell marker gene expression in human adipose tissue highly correlate in patients with obesity

**Fig 3:**
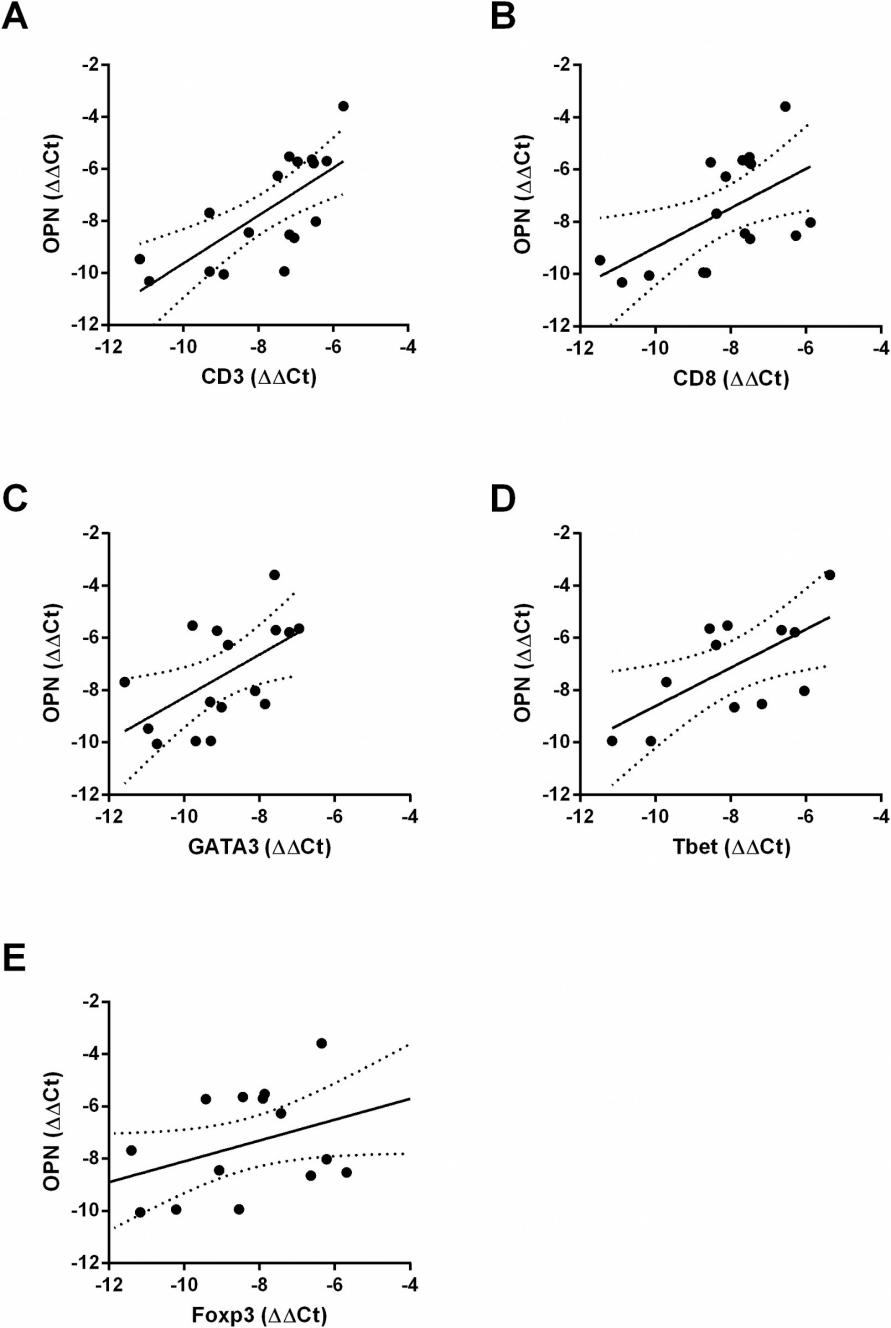
CD3, CD8 and GATA3 correlate significantly with OPN gene expression in human adipose tissue. Omental adipose tissue from individuals with obesity was isolated and (A) CD3 and OPN gene expression were analyzed by RT-PCR (n = 17) Spearman coefficient: r = 0.723, p = 0.0005 (B) CD8 and OPN gene expression (n = 17): r = 0.5637, p = 0.0203; (C) GATA3 and OPN gene expression (n = 16): r = 0.5206, p = 0.04; (D) Tbet and OPN (n = 12): r = 0.4825, p = 0.1154; (E) Foxp3 and OPN (n = 16): r = 0.3971, p = 0.1289; * p < 0.05, ** p < 0.01, *** p < 0.001 and **** p < 0.0001. Best fit line as well as the 95% confidence interval are indicated by lines in the graphs.

To validate these murine data in humans, we investigated potential correlation of OPN levels and T cell numbers as estimated by CD3 gene expression in omental adipose tissue from obese individuals. Obesity leads to upregulation of CD3 gene expression [19] and OPN gene expression[6] in visceral adipose tissue. Comparison of OPN with CD3 expression measured by PCR showed (Fig 3A) that there was a highly significant correlation between CD3 and OPN gene expression (Spearman r = 0.6901; p = 0.0005) in omental adipose tissue from people with obesity. Similarly, CD8 and OPN gene expression (Fig 3B) correlated significantly (Spearman r = 0.5637; p = 0.0203) as did GATA3 and OPN (Fig 3C; Spearman r = 0.5206, p = 0.04). Tbet and Foxp3 expression showed a trend towards correlation with OPN expression (Fig 3D and 3E).

### FL-OPN and cleaved OPN promote the number of viable T cells

**Fig 4:**
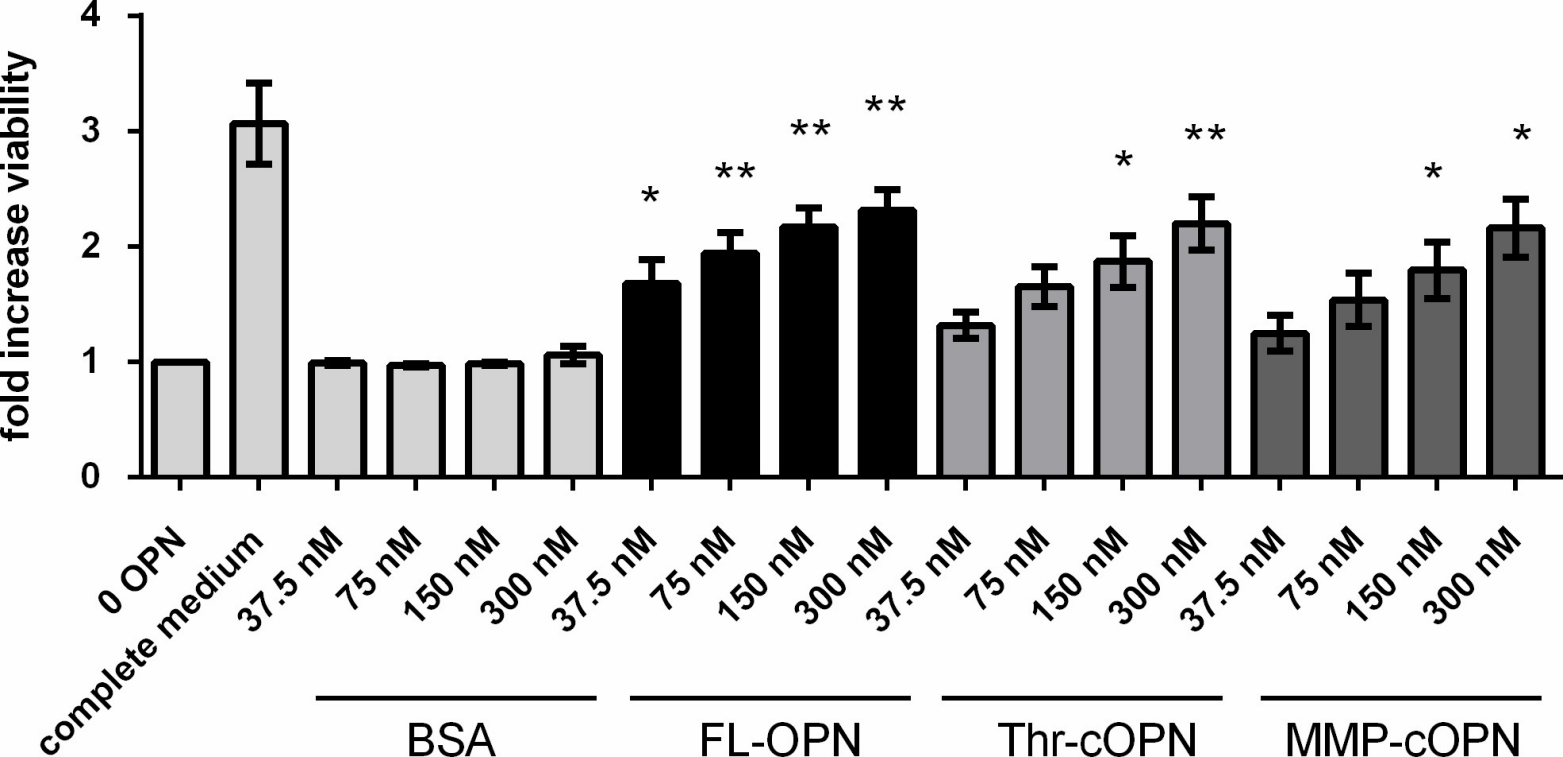
FL-OPN, Thr-cOPN and MMP-cOPN increase the number of viable T cells. Peripheral T cells (n = 3, 3 independent experiments) were incubated with FL-OPN, Thr-cOPN or MMP-cOPN under serum free conditions for 72 h. Release of ATP was assessed and ascribed to viable cells. Data was calculated by normalizing to the values without OPN (negative control). Complete medium included 10% FCS; mean ± SEM depicted; Comparison of OPN treated groups with the respective BSA control; * p < 0.05, ** p < 0.01.

To gain more information on how OPN might affect the numbers of T cells in the adipose tissue, its impact on their viability was investigated. In adipose tissue, full length OPN (FL-OPN) and cleaved OPN coexist [11]. FL-OPN, Thr-cOPN and MMP-cOPN were tested for their ability to promote the number of viable T cells. Primary human T cells were incubated with FL-OPN, Thr-cOPN and MMP-cOPN in serum free medium for 72 hours. Then release of ATP was measured as an indicator of cell viability. All tested forms of OPN increased the number of viable T cells in a dose-dependent manner with an EC_50_ of ~150nM and no significant differences between forms of OPN (Fig 4). Interestingly, OPN-CTF also promoted the viability of T cells (S2 Fig). As expected the positive control, cultivation of the cells in serum containing medium, also increased the number of viable cells over the BSA negative controls.

### T cells bind to full length-OPN and cleaved forms of OPN

**Fig 5:**
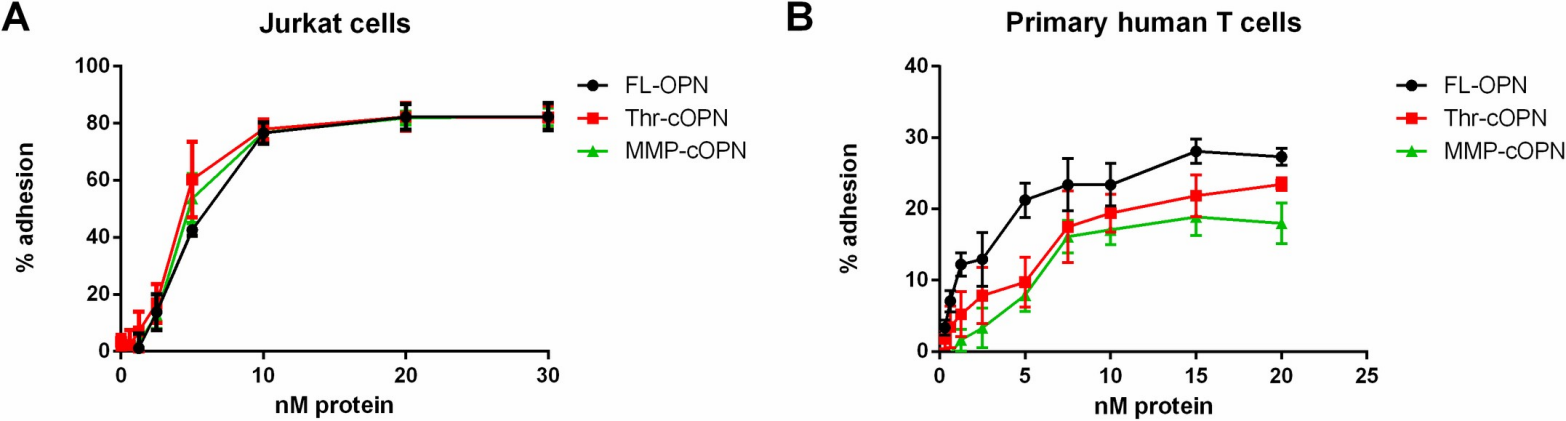
T cells bind to OPN. (A) Jurkat cells (2 experiments, each run in duplicate) and (B) primary T cells (4 experiments utilizing 4 donors, each run in duplicate) were analyzed for their adhesion to FL-OPN, Thr-cOPN and MMP-cOPN.

T cells bind to OPN via integrins with the extent of cell adhesion depending on the amount of matrix protein, the level of integrin expression on the T cells and the integrin activation state [24]. The reduced T cell marker gene expression in *Spp1*^*-/-*^ mice fed HFD might be the result of reduced infiltration of T cells or due to a reduction in T cell viability. In order to reduce inflammation by decreasing the number of T cells in adipose tissue, we explored an immunotherapeutic approach that targets the OPN-T cell interaction. In order to validate the assay in the absence of antibodies [25,26], we employed an adhesion assay to investigate the OPN-T cell interaction using primary human T cells or Jurkat cells. Around 80% of the input Jurkat cells (Fig 5A) bound to FL-OPN, Thr-cOPN or MMP-cOPN compared to around 25% of primary human T cells (Fig 5B). Cleavage of OPN by thrombin or MMP, which exposes the C-terminal VLA4 integrin-binding SVVYGLR and SVVYG sequences respectively, did not result in enhanced adhesion of T cells to OPN[27].

### OPN-T cell interactions are antagonized by anti-OPN antibodies

**Fig 6:**
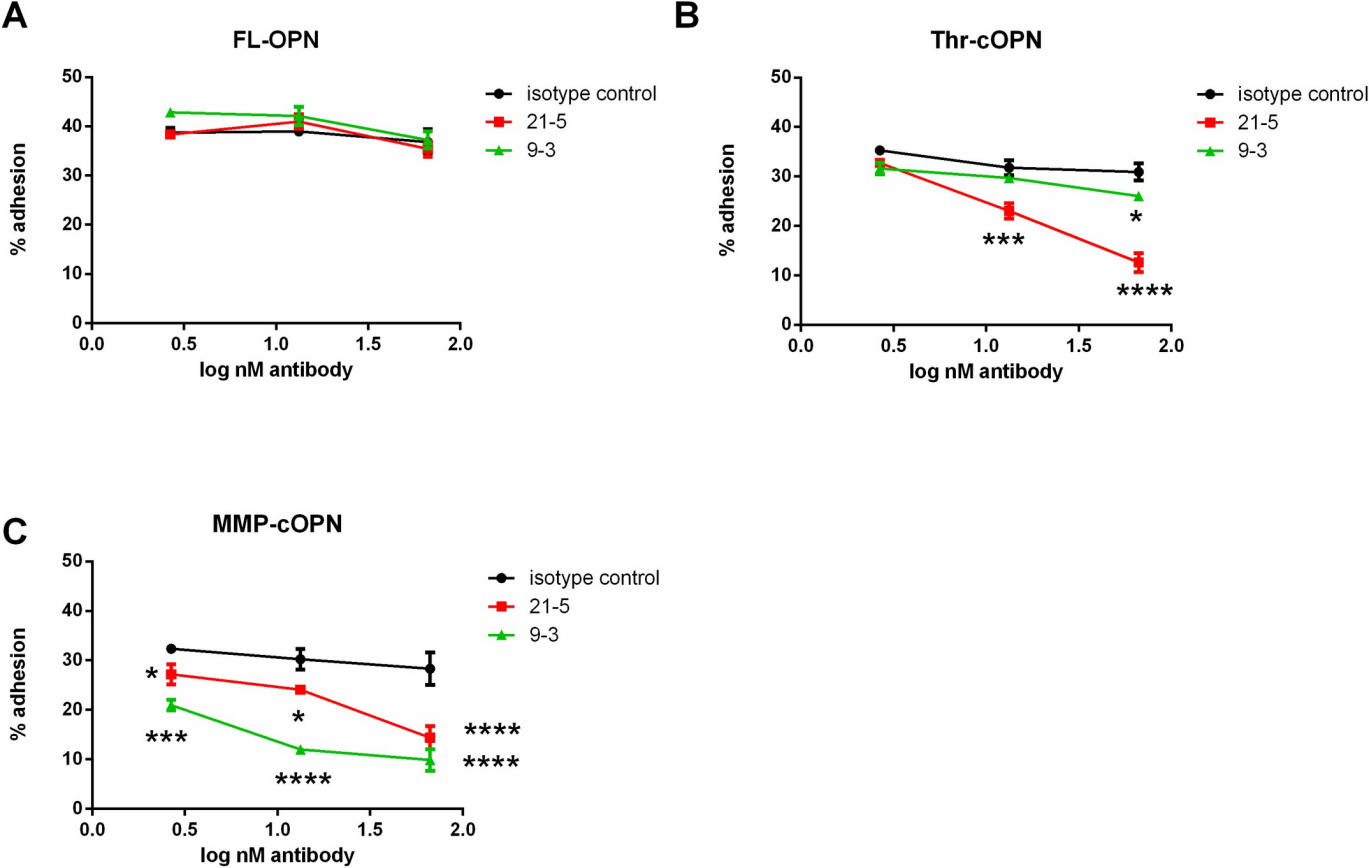
The interaction between T cells and cleaved OPN can be blocked with monoclonal antibodies. (A-C) Blockade of the interaction of primary human T cells with OPN with monoclonal antibodies 21–5 and 9–3. 1 experiment, 2 donors, each run in duplicate (A) Blockade of T cell binding to FL-OPN. (B) Blockade of T cell binding to Thr-cOPN. (C) Blockade of T cell binding to MMP-cOPN; mean ± SEM depicted; Comparison with the isotype control antibody; * p < 0.05, ** p < 0.01 and *** p < 0.001; **** p < 0.0001.

Cleavage of OPN by thrombin or MMPs occurs during inflammation, with cleaved OPN having different properties from FL-OPN. We developed Mabs with the aim of reducing inflammation without affecting other OPN-FL functions. As a first step towards demonstrating reduction of T cell infiltration without compromising other OPN functions we tested the effects of anti-OPN antibodies on adhesion by investigating their ability to block T cell binding to FL-OPN, Thr-cOPN and MMP-cOPN (Fig 6A–6C). The antibodies markedly inhibited the interaction between T cells and cleaved forms of OPN but not binding to FL-OPN. Antibody 21–5 was more potent in antagonizing T cell binding to Thr-cOPN and MMP-cOPN, while antibody 9–3 was more potent in inhibiting their binding to MMP-cOPN, with neither Mab affecting binding to FL-OPN. The preference of antibody 21–5 towards Thr-cOPN rather than MMP-cOPN may be explained by the fact that the peptide antigen against which the antibody was raised (C-TYDGRGDSVVYG-CO-NH2) contains a C-terminal amide group, which masks the carboxylic acid that is generated by MMP cleavage site.

**Fig 7:**
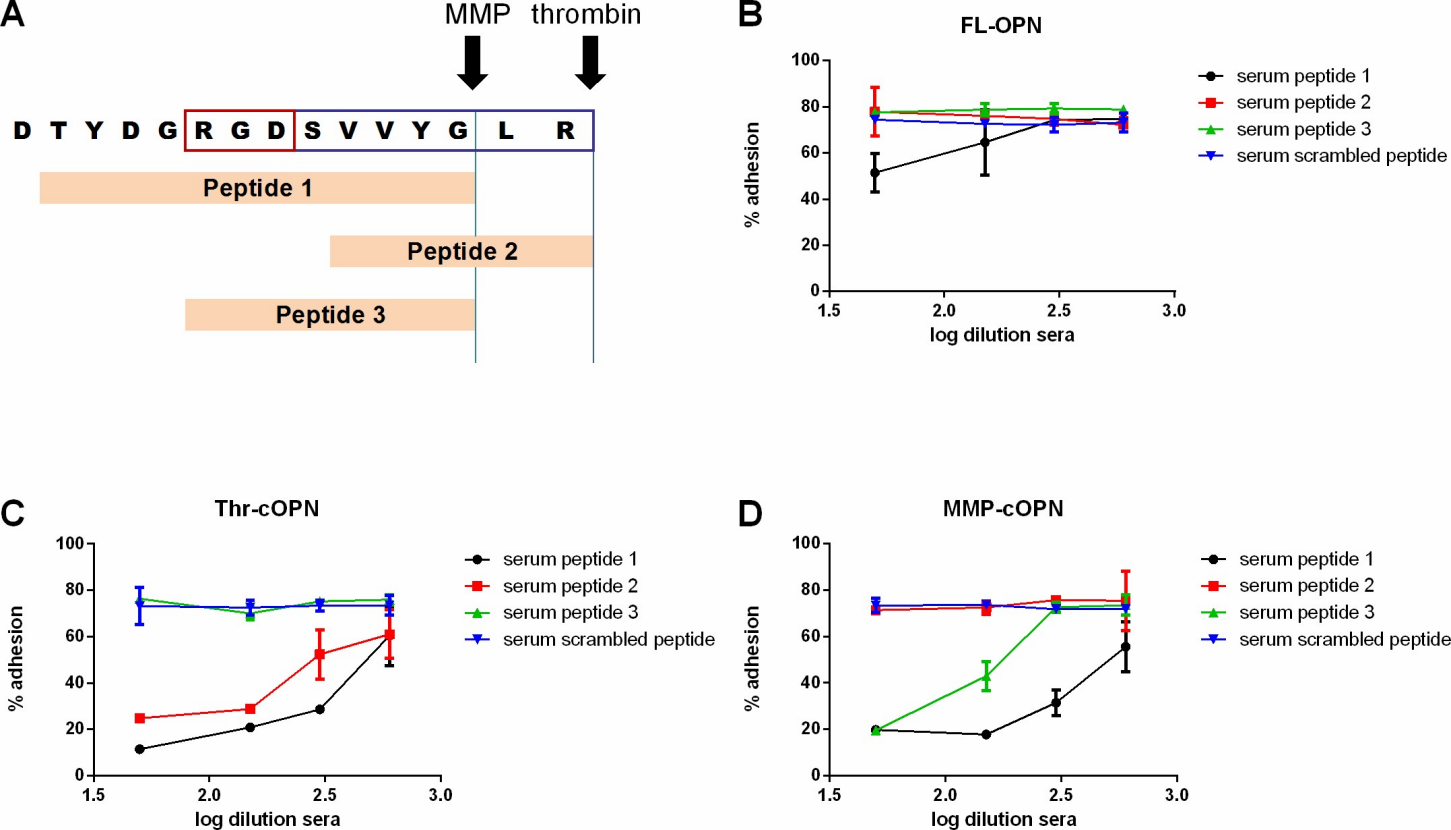
The OPN-T cell interaction can be antagonized by serum from active vaccination with OPN peptides. (A) 3 peptides derived from the OPN sequence plus a scrambled peptide were injected into mice. The MMP and thrombin cleavage sites are delineated by the vertical lines. The RGD binding site for α_v_β_3_ integrins is marked with a red box and the SVVYGLR binding site for α_4_β_1_ and α_4_β_9_ integrins is marked with a mauve box. (B) Interference of postimmune serum with binding of Jurkat T cells to FL-OPN. (C) Interference of postimmune serum with binding of Jurkat T cells to Thr-cOPN. (D) Interference of postimmune serum with Jurkat T cells binding to MMP-cOPN; The different sera for the experiments were pooled from 5 mice with the OPN-T cell interaction determined once with each point detected in duplicate; mean ± SD depicted.

We also investigated if an active vaccination strategy to target OPN-T cell interactions was feasible by testing the antisera from animals vaccinated with OPN-peptides (Fig 7A) for their ability to prevent T cell binding to OPN. These antisera successfully interfered with OPN-T cell interactions in a selective manner (Fig 7B–7D). Antibodies raised against peptide 1, TYDGRGDSVVYG, interfered with T cell binding to Thr-cOPN, MMP-cOPN and to a limited extent, FL-OPN. Peptide 2, VVYGLR, elicited antisera that only blocked T cell binding to Thr-cOPN while peptide 3, RGDSVVYG, elicited antibodies that interfered with T cell binding to MMP-cOPN. Thus it is possible to raise sera by active immunization that discriminate between these forms of cleaved OPN and FL-OPN.

## Discussion

Because OPN increases the number of macrophages in adipose tissue in obesity [6], we hypothesized that OPN might also influence the number of T cells, which precede macrophage accumulation in adipose tissue. To this end, we compared the level of T cells in adipose tissue from wt and *Spp1*^*-/-*^ obese mice. *Spp1*^*-/-*^ mice on LFD were not included in this study as they do not show any systemic or adipose tissue related change compared to wt mice on LFD. Also both plasma inflammatory cytokine levels and inflammatory cytokine mRNA expression in adipose tissue are the same in wt mice and *Spp1*^*-/-*^ mice on LFD. [6,16]. The significant increase in adipose tissue gene expression of the T cell markers, CD3, CD4 and CD8, in wt mice indicated T cell enrichment in adipose tissue in obesity confirming previous studies [1–3]. In contrast, OPN deficient *Spp1*^*-/-*^ mice had reduced T cell marker gene expression demonstrating that presence of OPN is critical for T cell accumulation in obese adipose tissue. We could further corroborate this result by staining for CD3, CD4 and CD8 markers in adipose tissue by immunofluorescence where we found that the signal intensity in wt mice fed HFD was significantly higher than in HFD *Spp1*^*-/-*^ mice suggesting that OPN is implicated in the increased number of T cell numbers in adipose tissue.

Of note, in our study, *Spp1*^*-/-*^ mice and wt mice on HFD had the same weight, which is critically important when comparing adipose tissue inflammation. Several authors have described that deficiency of *Spp1* (OPN) does not affect weight in comparison to wt mice [6,15,16]. In contrast, however, under certain circumstances, *Spp1*^*-/-*^ mice might gain less weight on HFD compared to wt mice, maybe due to use of mouse chow that was not nutrient adjusted between HFD and LFD [28]. Furthermore, the strong correlation between OPN and CD3 expression in individuals with obesity not only underlines the importance of OPN for T cell accumulation in obesity but extends this finding to humans. [1–3,19].

In addition to secreted OPN there is also an intracellular form of OPN (iOPN)[29]. Both forms are translated from the same mRNA and so PCR will not distinguish them. As iOPN is not present in the external environment, our hypothesis is that only extracellular OPN affects recruitment of T cells and this pool alone will be affected by administration of antibodies in cell-based assays or *in vivo*.

Presence of OPN in adipose tissue has been shown on the gene [6,16,30] and protein level by immunohistochemistry [30]. In obesity, proteolytic activity is present in the inflammatory environment of adipose tissue [11,31,32]. Consequently not only FL-OPN, but also cleaved forms of OPN are present in adipose tissue with MMP-cleaved OPN being more pro-inflammatory and modulating adipocyte function than FL-OPN [11]. It has not been shown yet whether Thr-cOPN [33] is present in adipose tissue. However, obese mice strongly express tissue factor in adipose tissue [34] suggesting that the coagulation cascade is activated in situ leading to an activation of thrombin which results in a significant local generation of Thr-cOPN.

OPN is not only involved in tissue infiltration by inflammatory cells [20,35,36]. We show here that FL-OPN [37] and its cleaved forms Thr-cOPN and MMP-cOPN increase T cell viability. In addition, we showed that the C-terminal fragment resulting from thrombin cleavage of OPN also increases T cell viability. Thus, interestingly, different regions of the molecule may induce survival signals which is worth to be further investigated. This might significantly contribute to T cell accumulation in adipose tissue. The OPN concentrations chosen for the viability assay were relatively high (up to 300 nM) to see the maximum response. In humans, the plasma levels of OPN have been reported to be around 400 ng/ml [38], corresponding to a concentration of roughly 9 nM. As OPN is a matricellular protein, the local concentration around OPN secreting cells might be much higher.

As OPN binding to T cells is a prerequisite for their modulation, we were interested in developing an immunotherapeutic approach that targets the OPN-T cell interaction to reduce T cell numbers in adipose tissue in order to reduce adipose tissue inflammation. To identify antibodies and antisera that block the OPN-T cell interaction, an adhesion assay using Jurkat cells or primary human T cells was established [20]. Binding of either Jurkat or primary T cells to OPN was not increased if OPN had been cleaved supporting earlier data [27], but in contrast with another study [23], most probably due to different experimental conditions.

Effective therapies are needed for metabolic syndrome and other disorder linked to obesity-driven inflammation. In a novel therapeutic approach, passive vaccination with an anti-OPN antibody reduced insulin resistance in a DIO mouse model[5] prompting us to develop antibodies recognizing human Thr-cOPN and MMP-cOPN. We identified antibodies that could block interactions between both Thr-cOPN and MMP-cOPN and primary human T cells. These antibodies recognize only cleaved forms of OPN, demonstrating that it is possible to develop Mabs targeting these OPN forms that are generated during inflammation. This may allow specifically blocking OPN functions uncovered upon protease cleavage while leaving physiological functions of FL-OPN unaffected.

However, passive vaccination suffers from the problem of the short half-lives of the antibodies–usually less than one month, requiring repeat administrations of the antibody. Thus making this approach both expensive and conferring a risk of poor treatment adherence. Additionally, anti-drug-antibodies might be elicited leading to reduced efficacy which is only prevented by use of fully human antibodies [39]. In contrast, active vaccination is by far less expensive and might induce a longstanding immune response. With an active vaccination approach presented here, antisera can be produced containing antibodies that specifically recognize the cleaved forms of OPN without binding to FL-OPN. An initial attempt using murine OPN-peptide-KLH conjugates, however, failed to induce OPN-specific antibodies[40]. In the work described in this paper, human OPN sequences were used and their human *in vivo* effects have not been elucidated yet.

Recently, a population of senescent CD4^+^ T cells has been described in adipose tissue [41,42] that are CD153^+^ PD-1^+^ CD44^hi^ and capable of secreting high amounts of OPN and thereby promoting adipose tissue inflammation. Adoptive transfer of these cells from wt mice fed HFD into gonadal fat of lean mice fed a normal diet, sharply elevated *Spp1* gene expression, promoted adipose tissue inflammation and reduced insulin sensitivity. Elimination of such senescence cells is not easily achieved. Therefore our strategy might make it possible to reduce the infiltration of T cells that then senesce in visceral fat. Targeting the OPN-T cell interaction is a promising approach as their interaction is a prerequisite for the subsequent migration of T cells into tissue.

In summary, OPN regulates the number of T cells in adipose tissue at least in part by promoting T cell viability. In addition, possibly OPN and its proteolytic cleavage fragments might also promote the migration of T cells into adipose tissue. These two mechanisms could explain the strong correlation between T cell markers and OPN found in adipose tissue of individuals with obesity. Moreover, the interaction between T cells and cleaved forms of OPN can be specifically blocked with specific monoclonal antibodies and post immune sera. The development of a successful active vaccination approach is hence warranted tackling initial adversities.

## Supporting information

S1 Fig Obese *Spp1*^*-/-*^ mice have less T cells in their gonadal fat than wt mice. Representative stainings of CD3, CD4, CD8 by immunofluorescence. CD3 and CD4 specific staining is shown in green. CD8 specific staining is shown in red. Large image: 10x objective with scale bar, inserts: 40x magnification.
(TIF)

S2 Fig The OPN-CTF promotes the viability of T cells. T cells were isolated from blood (n = 3 donors) and co-cultured with FL-OPN or OPN-CTF for 72 h under serum free condition. 300 nM BSA was used as a negative control. ATP levels were taken as a measure for cell viability. mean ± SEM depicted; Comparison of OPN treated groups with the BSA control; * p < 0.05, ** p < 0.01, *** p < 0.001, **** p < 0.0001.
(TIF)
