# Supplementary figures and images for: Antibody-mediated targeting of cleavage-specific OPN-T cell interactions
Source: PLoS One. 2019 Apr 5;14(4):e0214938. doi: 10.1371/journal.pone.0214938 (PMC6450625; doi:10.1371/journal.pone.0214938)

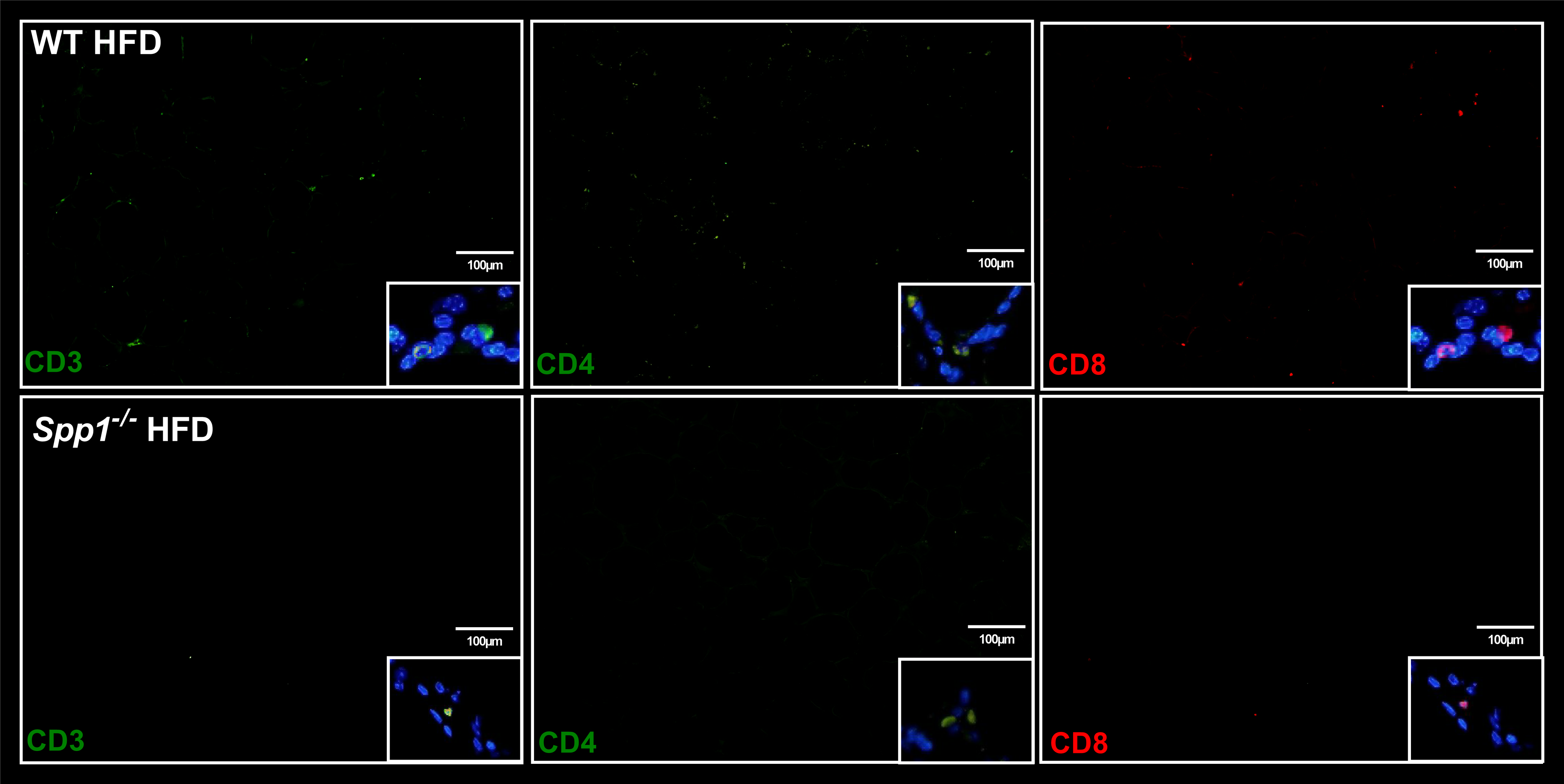

Supplement: S1 Fig — Representative stainings of CD3, CD4, CD8 by immunofluorescence. CD3 and CD4 specific staining is shown in green. CD8 specific staining is shown in red. Large image: 10x objective with scale bar, inserts: 40x magnification. (TIF) [file pone.0214938.s001.tif]

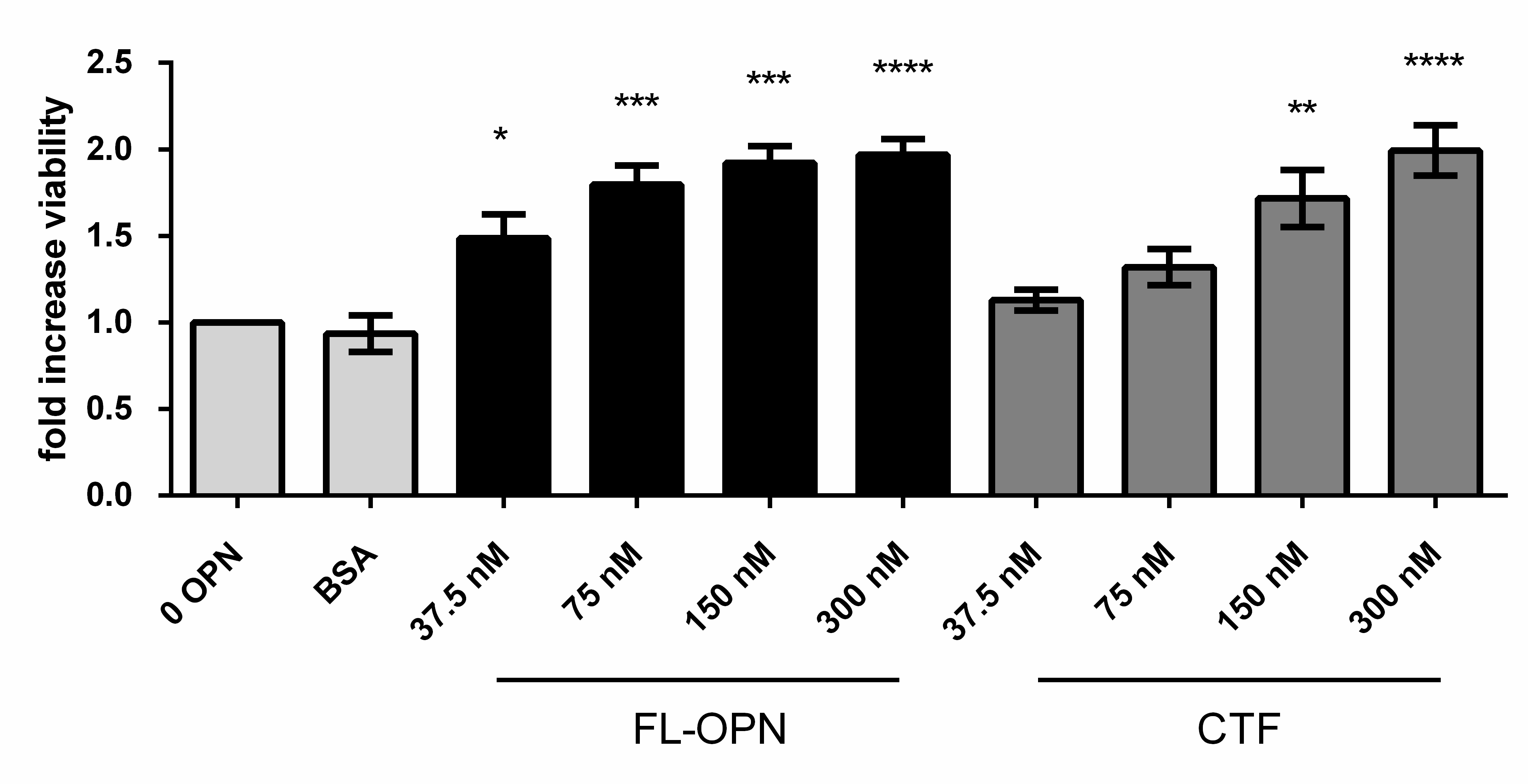

Supplement: S2 Fig — T cells were isolated from blood (n = 3 donors) and co-cultured with FL-OPN or OPN-CTF for 72 h under serum free condition. 300 nM BSA was used as a negative control. ATP levels were taken as a measure for cell viability. mean ± SEM depicted; Comparison of OPN treated groups with the BSA control; * p < 0.05, ** p < 0.01, *** p < 0.001, **** p < 0.0001. (TIF) [file pone.0214938.s002.tif]
